# Enzyme replacement therapy in mice lacking arylsulfatase B targets bone-remodeling cells, but not chondrocytes

**DOI:** 10.1093/hmg/ddaa006

**Published:** 2020-01-15

**Authors:** Gretl Hendrickx, Tatyana Danyukova, Anke Baranowsky, Tim Rolvien, Alexandra Angermann, Michaela Schweizer, Johannes Keller, Jörg Schröder, Catherine Meyer-Schwesinger, Nicole Muschol, Chiara Paganini, Antonio Rossi, Michael Amling, Sandra Pohl, Thorsten Schinke

**Affiliations:** 1 Department of Osteology and Biomechanics, University Medical Center Hamburg-Eppendorf, 20246 Hamburg, Germany; 2 Department of Electron Microscopy, Center of Molecular Neurobiology, University Medical Center Hamburg-Eppendorf, 20246 Hamburg, Germany; 3 Center for Musculoskeletal Surgery, Charité University Medicine, 10117 Berlin, Germany; 4 Institute of Cellular and Integrative Physiology, University Medical Center Hamburg-Eppendorf, 20246 Hamburg, Germany; 5 International Center for Lysosomal Diseases, University Medical Center Hamburg-Eppendorf, 20246 Hamburg, Germany; 6 Department of Molecular Medicine, University of Pavia, 27100 Pavia, Italy

## Abstract

Mucopolysaccharidosis type VI (MPS-VI), caused by mutational inactivation of the glycosaminoglycan-degrading enzyme arylsulfatase B (Arsb), is a lysosomal storage disorder primarily affecting the skeleton. We have previously reported that Arsb-deficient mice display high trabecular bone mass and impaired skeletal growth. In the present study, we treated them by weekly injection of recombinant human ARSB (rhARSB) to analyze the impact of enzyme replacement therapy (ERT) on skeletal growth and bone remodeling. We found that all bone-remodeling abnormalities of Arsb-deficient mice were prevented by ERT, whereas chondrocyte defects were not. Likewise, histologic analysis of the surgically removed femoral head from an ERT-treated MPS-VI patient revealed that only chondrocytes were pathologically affected. Remarkably, a side-by-side comparison with other cell types demonstrated that chondrocytes have substantially reduced capacity to endocytose rhARSB, together with low expression of the mannose receptor. We finally took advantage of Arsb-deficient mice to establish quantification of chondroitin sulfation for treatment monitoring. Our data demonstrate that bone-remodeling cell types are accessible to systemically delivered rhARSB, whereas the uptake into chondrocytes is inefficient.

## Introduction

The skeleton is a highly complex and dynamic tissue consisting of more than 200 differently shaped elements. Skeletal development is grossly divided into two types of ossification, i.e. intramembranous or endochondral (1). Intramembranous ossification occurs primarily in the skull bones, where mesenchymal progenitor cells directly differentiate into bone-forming osteoblasts (2). The majority of skeletal elements, including the long bones, develop by endochondral ossification, where a cartilage intermediate is first formed (3). More specifically, mesenchymal cells condensate to form chondrocytes, which undergo further differentiation into hypertrophic cells producing a mineralized cartilage matrix, which is subsequently replaced by bone (4). This initial bone formation step, occurring in the center of the developing skeletal element, generates two zones, where the chondrocytes continue to differentiate into hypertrophic cells from both sides. These zones, termed growth plates, are composed of chondrocytes undergoing a coordinated differentiation program required for skeletal growth (5). During development and growth, but also thereafter, the bone matrix is continuously remodeled by two different cell types, bone-resorbing osteoclasts and bone-forming osteoblasts. Bone remodeling, which is required for long-term skeletal integrity, is controlled, at least in part, by osteocytes, which are matrix-embedded terminally differentiated osteoblasts (6). These complexities of skeletal development, growth and remodeling, which involve several distinct cell types, explain the importance of deep-phenotyping approaches to fully uncover specific cellular deficits in different disorders.

One disease with remarkable, yet poorly understood influence on the skeleton is mucopolysaccharidosis type VI (MPS-VI, OMIM 253200). This lysosomal storage disorder is caused by mutational inactivation of arylsulfatase B (Arsb), which is required to facilitate one critical step in the lysosomal degradation of chondroitin and dermatan sulfate (7). Similar to other forms of MPS, the lack of Arsb activity results in lysosomal accumulation of non-degraded glycosaminoglycans, which are also increased in the urine. Importantly, however, unlike in patients with MPS-I or MPS-III, characterized by impaired lysosomal degradation of heparan sulfate, there are no neurological symptoms associated with MPS-VI (8,9). In fact, the respective patients are mostly affected by various defects of skeletal development, growth and integrity, typically summarized as dysostosis multiplex (10). These defects, all well-documented radiologically, include short stature, enlargement of skull bones, hip dysplasia, joint stiffness and spinal cord compression. Besides additional complications of the musculoskeletal system, there are also impaired pulmonary function, hepatosplenomegaly and cardiac valve abnormalities in patients with MPS-VI, altogether causing decreased life expectancy (11).

At present, MPS-VI is mostly treated by enzyme replacement therapy (ERT), i.e. weekly infusion of the recombinant human ARSB (rhARSB) modified with mannose 6-phosphate (M6P) residues to allow M6P receptor-mediated cellular uptake and lysosomal delivery (12,13). This treatment causes significant reduction in urinary glycosaminoglycan levels and improves some established parameters of musculoskeletal and pulmonary functions (14). On the other hand, the major features of dysostosis multiplex are not efficiently corrected by ERT in patients with MPS-VI, which is often explained by failure of rhARSB to reach skeletal cell types via the circulation (15). Importantly, however, whereas cartilage is indeed a poorly vascularized tissue, the opposite is the case for bone (16). Therefore, it is highly relevant to understand the efficacy of ERT for the treatment of MPS-VI at the level of specific skeletal cell types, which can be achieved by bone-specific histomorphometry taking advantage of animal models.

In an unbiased screen for lysosomal enzymes with differential expression during osteoclastogenesis, we have previously identified Arsb, which led us to study the bone phenotype of a mouse model carrying an inactivating *Arsb* mutation (thereafter termed *Arsb^m/m^* mice). We found that *Arsb^m/m^* mice display various features of dysostosis multiplex, including reduced skeletal growth, as well as lysosomal storage in all relevant skeletal cell types (17). Analysis of the bone-remodeling phenotype revealed that 12-week-old *Arsb^m/m^* mice display moderate osteopetrosis, i.e. high trabecular bone mass due to impaired osteoclast function (17,18). Importantly, this phenotype was fully corrected, when the mice were treated by ERT from 12 to 24 weeks of age. This experiment, however, did not address the question, if ERT is sufficient to improve chondrocyte-related defects, such as impaired skeletal growth, since this requires treatment initiation in growing mice. Therefore, the aim of the present study was to define the skeletal phenotype of 12- and 24-week-old *Arsb^m/m^* mice, where treatment by ERT was initiated at 4 weeks of age.

## Results

### ERT of Arsb-deficient mice prevents development of a high bone mass phenotype, but does not normalize skeletal growth

**Figure 1:**
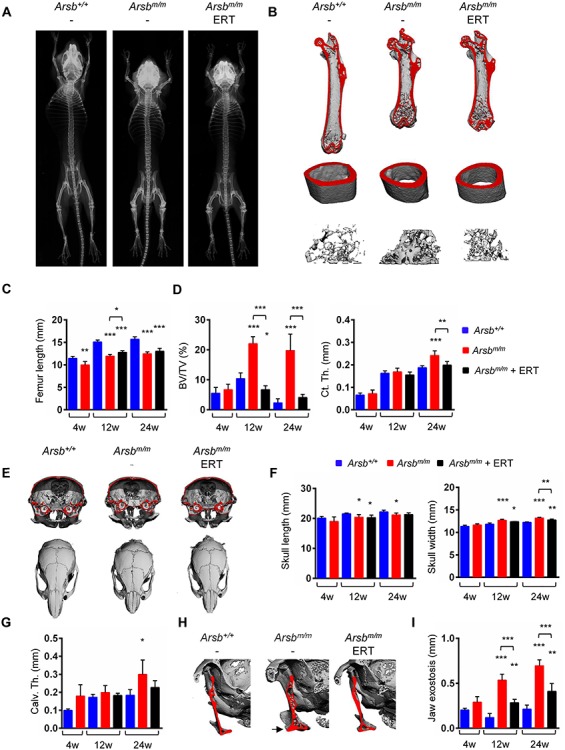
ERT of Arsb-deficient mice affects bone mass, but not skeletal growth. (**A**) Representative contact Xray images from 24-week-old wild-type (*Arsb^+/+^*), untreated (*Arsb^m/m^*) or treated (*Arsb^m/m^* ERT) Arsb-deficient mice. (**B**) Representative μCT images of femora from the same groups of animals (top, total femur; middle, midshaft cortical bone; bottom, trabecular bone in the proximal femur). (**C**) Quantification of femur length in wild-type (blue bars), untreated (red bars) or treated (black bars) Arsb-deficient mice at 4, 12 or 24 weeks of age. (**D**) Quantification of trabecular bone mass (BV/TV) and of cortical thickness (Cort. Th.) in the same mice (*n* ≥ 5 mice per group). (**E**) Representative μCT images of skull bones from 24-week-old wild-type (*Arsb^+/+^*), untreated (*Arsb^m/m^*) or treated (*Arsb^m/m^* ERT) Arsb-deficient mice (top, cross-sectional; bottom, top view). (**F**) Quantification of skull length (left) and width (right) in wild-type (blue bars), untreated (red bars) or treated (black bars) Arsb-deficient mice at 4, 12 or 24 weeks of age (*n* ≥ 5 mice per group). (**G**) Quantification of calvarial thickness (Calv. Th.) in the same mice (*n* ≥ 5 mice per group). (**H**) Representative μCT images of jaw bones from 24-week-old wild-type (*Arsb^+/+^*), untreated (*Arsb^m/m^*) or treated (*Arsb^m/m^* ERT) Arsb-deficient mice (*n* ≥ 5 mice per group). (**I**) Quantification of jaw exostoses (indicated by the arrow) in wild-type (blue bars), untreated (red bars) or treated (black bars) Arsb-deficient mice at 4, 12 or 24 weeks of age (*n* ≥ 5 mice per group). Data are expressed as mean ± SD. ^*^P < 0.05, ^**^P < 0.005, ^***^P < 0.0005 (one-way ANOVA).

For the present study, we took advantage of the *Arsb^m/m^* mice as a model of MPS-VI and treated them by weekly injection of rhARSB (ERT) starting at 4 weeks of age. We then analyzed the skeletal phenotypes of 12- and 24-week-old animals (i.e. after 8 or 20 weeks of treatment, respectively), which were compared to untreated wild-type and *Arsb^m/m^* littermate controls. Based on the initial contact X-rays it was evident that the long-term treatment by ERT did not increase the length of skeletal elements in *Arsb^m/m^* mice, whereas their high bone mass phenotype appeared to be normalized (Fig. 1A). This conclusion was fully supported by μCT imaging of the femoral bones (Fig. 1B). Quantification of the respective data in the three groups of mice confirmed that 12- and 24-week-old *Arsb^m/m^* mice displayed significantly reduced length of the femoral bones, either with or without treatment (Fig. 1C). In contrast, ERT profoundly affected the high trabecular bone mass phenotype of *Arsb^m/m^* mice. This was particularly evident in the older group of mice, where the trabecular bone volume per tissue volume (BV/TV) was more than 5-fold higher in untreated *Arsb^m/m^* mice, but not in treated *Arsb^m/m^* mice (Fig. 1D). Of note, 24-week-old *Arsb^m/m^* mice additionally displayed increased cortical thickness in the femoral midshaft region, which was prevented by ERT.

**Figure 2:**
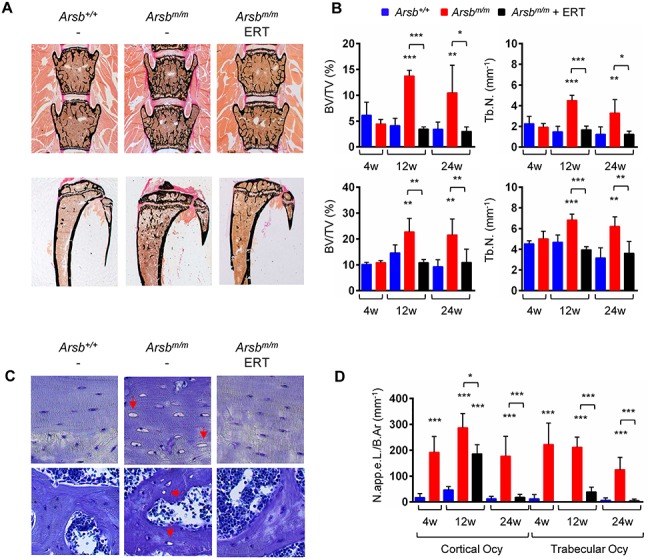
ERT prevents bone-remodeling pathologies in Arsb-deficient mice. (**A**) Representative undecalcified spine (top) and tibia (bottom) sections from 24-week-old wild-type (*Arsb^+/+^*), untreated (*Arsb^m/m^*) or treated (*Arsb^m/m^* ERT) Arsb-deficient mice after von Kossa-van Gieson staining. Mineralized bone matrix appears black. (**B**) Quantification of trabecular bone mass (BV/TV) and Tb.N. in spine (top) and tibia (bottom) sections from wild-type (blue bars), untreated (red bars) or treated (black bars) Arsb-deficient mice at 4, 12 or 24 weeks of age (*n* ≥ 5 mice per group). (**C**) Representative images showing cortical (top) and trabecular (bottom) sections from 24-week-old wild-type (*Arsb^+/+^*), untreated (*Arsb^m/m^*) or treated (*Arsb^m/m^* ERT) Arsb-deficient mice after toluidine blue staining. Enlarged and apparently empty osteocyte lacunae (indicated by the arrows) are specifically observed in untreated *Arsb^m/m^* mice. (**D**) Quantification of apparently empty osteocyte lacunae (N.app.e.L./B.Ar., number of apparently empty lacunae per bone area) in cortical and trabecular bone from wild-type (blue bars), untreated (red bars) or treated (black bars) Arsb-deficient mice at 4, 12 or 24 weeks of age (*n* ≥ 5 mice per group). Data are expressed as mean ± SD. ^*^P < 0.05, ^**^P < 0.005, ^**^P < 0.0005 (one-way ANOVA).

Since patients with MPS-VI typically display altered morphology and overgrowth of craniofacial bones, we additionally analyzed these skeletal elements in the different groups of mice by μCT imaging (Fig. 1E). We observed that *Arsb^m/m^* mice displayed reduced length and increased width of the skull, which was improved by ERT (Fig. 1F). However, since the differences towards wild-type littermates were rather moderate, the statistical analysis was not as informative as for the femoral parameters described above. Nevertheless, consistent with the cortical thickness in the femoral bones, there was an increased calvarial thickness in 24-week-old untreated *Arsb^m/m^* mice, which was not observed in the treatment group (Fig. 1G). Finally, as previously established as the major craniofacial abnormality of *Arsb^m/m^* mice (17), we determined the exostosis of jaw bones in the different groups (Fig. 1H). Here, we observed a positive treatment effect, although jaw exostosis was still increased in treated *Arsb^m/m^* mice compared to wild-type controls (Fig. 1I). Importantly, however, while there was a near 2-fold increase of jaw exostosis in untreated *Arsb^m/m^* mice between 4 and 12 weeks of age, this did not appear in mice receiving ERT. Taken together, these data reveal that MPS-VI-associated defects of bone-remodeling and intramembranous ossification are fully or partially prevented by ERT, respectively. In contrast, there was no positive effect of ERT on impaired skeletal growth, suggesting that chondrocyte defects are not corrected by the treatment.

### ERT prevents bone-remodeling pathologies in Arsb-deficient mice

To substantiate these findings at the cellular level, we analyzed undecalcified spine and tibia sections from the different groups of mice (Fig. 2A). Quantification of the trabecular bone volume (BV/TV) and trabecular number (Tb.N.) demonstrated the efficacy of ERT to prevent *Arsb^m/m^* pathologies. In particular, while both parameters were not different between wild-type and *Arsb^m/m^* littermates at 4 weeks of age, 12- and 24-week-old *Arsb^m/m^* animals displayed a high bone mass phenotype in the lumbar spine and in the tibia (Fig. 2B). Most importantly, this phenotype did not develop when the mice were treated by ERT. We have previously found that bone-embedded *Arsb^m/m^* osteocytes are severely affected by lysosomal storage and that this pathology is detectable on bone sections stained with toluidine blue (17). More specifically, while most osteocytes in *Arsb^m/m^* sections appear strikingly enlarged, there are also apparently empty osteocyte lacunae, where no nuclei are visible (Fig. 2C).

Since these occurred in trabecular and cortical bone, we took advantage of this readout as a parameter for osteocyte pathologies. Quantitative analysis of tibia sections revealed a significant increase of apparently empty osteocyte lacunae in both bone compartments of *Arsb^m/m^* mice, and this pathology was already pronounced at 4 weeks of age (Fig. 2D). Importantly, the number of apparently empty osteocyte lacunae was significantly reduced by ERT and fully normalized in both compartments in 24-week-old treated *Arsb^m/m^* mice. In 12-week-old animals, however, the ERT correction of trabecular osteocytes was more efficient than it was in cortical bone, which is likely explained by the higher rate of bone remodeling in the trabecular compartment (19). Taken together, these data demonstrate that systemic rhARSB delivery is sufficient to reach bone-remodeling cell types, which prevents and/or corrects their lysosomal storage.

### Chondrocyte pathologies in Arsb-deficient mice remain unaffected by ERT

**Figure 3:**
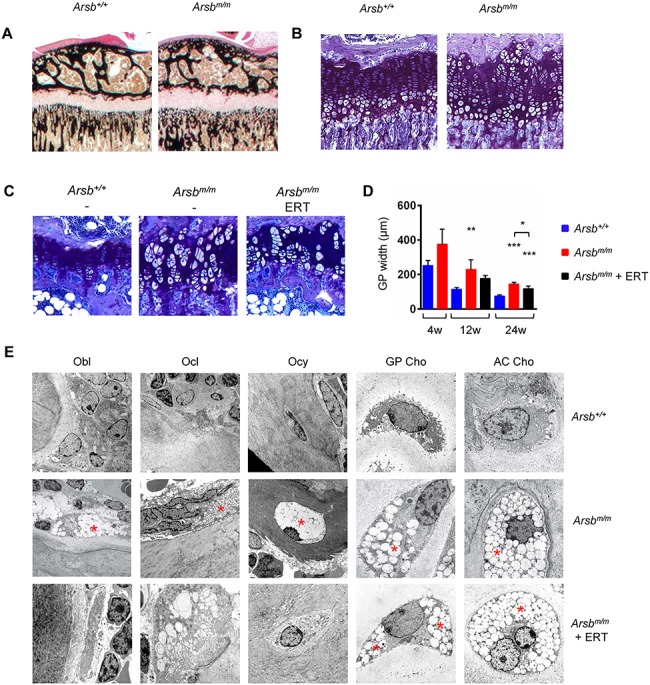
Chondrocyte pathologies in Arsb-deficient mice are not affected by ERT. (**A**) Representative images showing the width of the growth plates in undecalcified tibia sections from 4-week-old wild-type (*Arsb^+/+^*) and Arsb-deficient mice (*Arsb^m/m^*) after von Kossa-van Gieson staining. (**B**) Representative images showing the growth plates in tibia sections from the same animals after toluidine blue staining. (**C**) Representative images showing the growth plates in tibia sections from 24-week-old wild-type (*Arsb^+/+^*), untreated (*Arsb^m/m^*) or treated (*Arsb^m/m^* ERT) Arsb-deficient mice after toluidine blue staining. (**D**) Quantification of growth plate (GP) width in tibia sections from wild-type (blue bars), untreated (red bars) or treated (black bars) Arsb-deficient mice at 4, 12 or 24 weeks of age (*n* ≥ 5 mice per group). (**E**) Representative electron microscopy images showing osteoblasts (Obl), osteoclasts (Ocl), osteocytes (Ocy), and chondrocyte in the growth plate (GP Cho) or articular cartilage (AC Cho) from 12-week-old wild-type (*Arsb^+/+^*), untreated (*Arsb^m/m^*) or treated (*Arsb^m/m^* ERT) Arsb-deficient mice. Lysosomal storage is indicated by the asterisks. Data are expressed as mean ± SD. ^*^P < 0.05, ^**^P < 0.005, ^***^P < 0.0005 (one-way ANOVA).

We additionally analyzed the growth plate regions of undecalcified bone sections in the different groups of mice. In contrast to the tibia sections of 24-week-old *Arsb^m/m^* mice, there was only moderate growth plate widening observed at 4 weeks of age (Fig. 3A). However, as revealed by toluidine blue staining, there was an obvious enlargement of growth plate chondrocytes in *Arsb^m/m^* animals (Fig. 3B). This pathology became much more severe in older mice, which was not prevented by ERT (Fig. 3C). Since the striking impact of Arsb deficiency on growth plate chondrocytes did not specifically affect a distinct region, i.e. the resting, proliferative or hypertrophic zone, we quantified this pathology by measuring the growth plate width. More specifically, this parameter was significantly increased in 12- and 24-week-old *Arsb^m/m^* mice, and it was not normalized by ERT (Fig. 3D).

To substantiate our findings, we analyzed 12-week-old mice by electron microscopy to screen for lysosomal storage in the different skeletal cell types. In osteoblasts, osteoclasts, osteocytes and chondrocytes of untreated *Arsb^m/m^* mice, we found enlarged lysosomes containing electron-lucent non-degraded macromolecules, which is the characteristic feature of lysosomal storage disorders (Fig. 3E). Most importantly, there were no morphological abnormalities observed in osteoblasts, osteoclasts or trabecular osteocytes of ERT-treated *Arsb^m/m^* mice. In contrast, growth plate and articular chondrocytes were still characterized by severe lysosomal storage, demonstrating the absence of a treatment effect.

**Figure 4:**
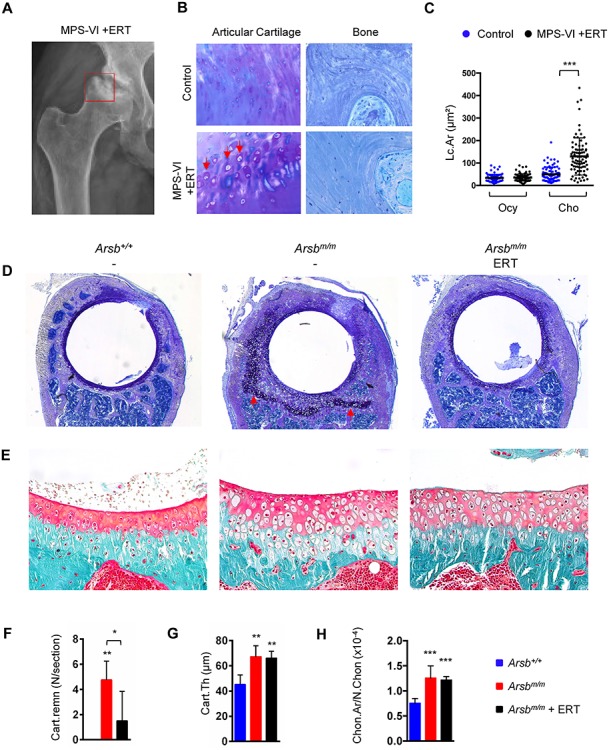
Articular chondrocyte pathologies are not prevented by ERT in an MPS-VI patient and in Arsb-deficient mice. (**A**) Hip radiograph of a 30-year-old female patient with MPS-VI, who received ERT for the last 4 years. The pathologically affected region (femoral head and acetabulum) is highlighted by the red square. (**B**) Representative images of articular cartilage (left) and bone (right) in bone biopsies of a control individual and the ERT-treated MPS-VI patient. Enlargement of articular chondrocytes is indicated by the arrows. (**C**) Quantification of the cellular size increase (Lc.Ar. lacunar area) in osteocytes (Ocy) and articular chondrocytes (Cho) in the different biopsies. (**D**) Representative images showing the acetabulum from 24-week-old wild-type (*Arsb^+/+^*), untreated (*Arsb^m/m^*) or treated (*Arsb^m/m^* ERT) Arsb-deficient mice after toluidine blue staining. The dark staining indicates the presence of cartilage remnants in untreated Arsb-deficient mice (arrows). (**E**) Representative images showing the articular regions from the same samples after Goldner staining. Note the enlarged cell sizes and increased cartilage thickness in untreated and treated Arsb-deficient mice. (**F**) Quantification of cartilage remnants (Cart.Remn.) in the acetabulum from wild-type (blue bars), untreated (red bars) or treated (black bars) Arsb-deficient mice at 24 weeks of age (*n* ≥ 5 mice per group). (**G**) Quantification of articular cartilage thickness (Cart.Th.) in the same samples (*n* ≥ 5 mice per group). (**H**) Quantification of articular chondrocyte size (Chon.Ar/N.Chon, chondrocyte area per number of chondrocytes) in the same samples (*n* ≥ 5 mice per group). Data are expressed as mean ± SD. ^*^P < 0.05, ^**^P < 0.005, ^***^P < 0.0005 (one-way ANOVA).

To confirm the human relevance of the respective findings, we histologically analyzed the femoral head that was surgically removed in a 30-year-old female patient with MPS-VI, who had been treated by ERT for the last 4 years (Fig. 4A). In comparison with a control biopsy, we found that the articular chondrocyte population was significantly enlarged in the patient, whereas the osteocyte population appeared morphologically normal (Fig. 4B). This observation was supported by quantification of the lacunar area within the stained regions of articular cartilage and bone, respectively (Fig. 4C). Since the requirement for orthopedic surgery in patients with MPS is typically attributed to defects of endochondral ossification in the acetabulum, we specifically analyzed the ERT effect on this skeletal element in the different groups of 24-week-old mice. Although there was greater variation compared to spine and tibia sections, it was remarkable that *Arsb^m/m^* mice displayed two major pathologies, i.e. cartilage remnants (Fig. 4D) and thickening of articular cartilage with enlarged chondrocytes (Fig. 4E). Consistent with the previously observed differential effects in other bones, the number of cartilage remnants, a typical sign of osteoclast dysfunction (20), was significantly reduced in *Arsb^m/m^* mice that received ERT (Fig. 4F). In contrast, the thickness of articular cartilage, as well as the size of articular chondrocytes, was not affected by the treatment (Fig. 4G). Taken together, these findings demonstrate that chondrocyte defects caused by Arsb inactivation are not prevented and/or corrected by systemic delivery of rhARSB, thereby raising the question about the underlying causes.

### Chondrocytes have decreased capacity to endocytose rhARSB

**Figure 5:**
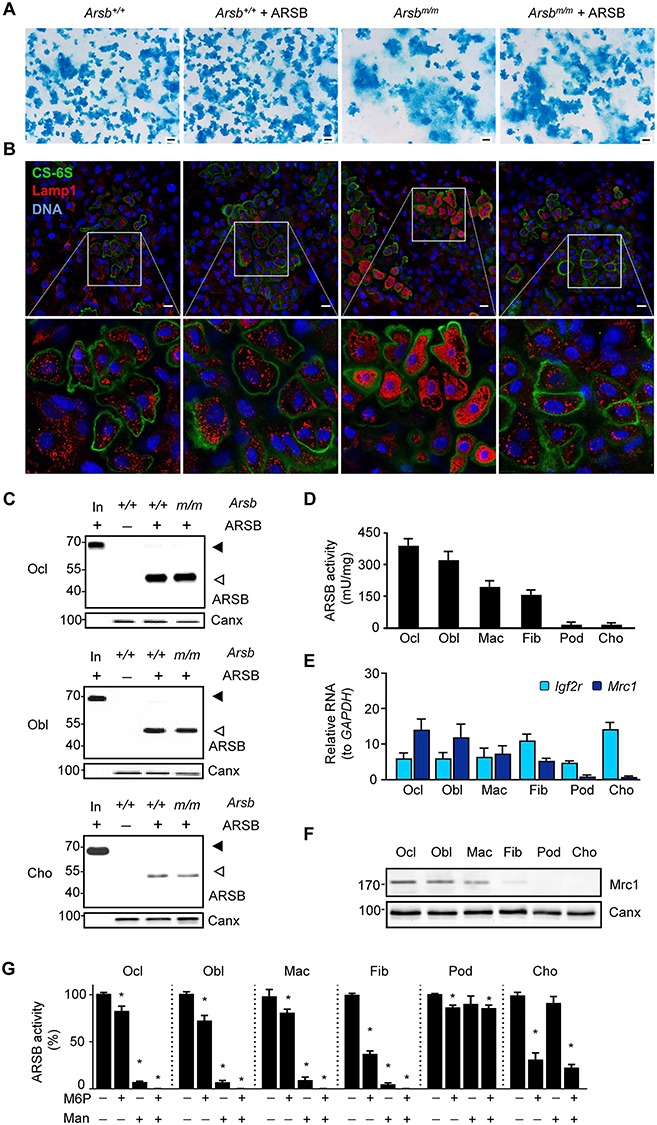
Chondrocytes have decreased capacity to endocytose rhARSB. (**A**) Representative images after alcian blue staining of cultured chondrocytes from wild-type (*Arsb^+/+^*) and Arsb-deficient (*Arsb^m/m^*) mice, cultured for 10 days in the absence or presence of ARSB, as indicated. (**B**) Immunostaining of the same cultures with antibodies against chondroitin 6-sulfate (green) and Lamp1 (red). Nuclei were visualized by DAPI stain (blue). (**C**) ARSB western blot of cell lysates from wild-type and Arsb-deficient osteoclasts (Ocl), osteoblasts (Obl) and chondrocytes (Cho) after incubation with ARSB for 4 h. Precursor (In, input) and mature forms of ARSB are indicated by closed or open arrowheads, respectively. Canx western blot was performed as loading control. (**D**) ARSB activity in cell lysates of different cell types (Ocl, osteoclasts; Obl, osteoblasts; Mac, macrophages; Fib, fibroblasts; Pod, podocytes; Cho, chondrocytes) after incubation with ARSB for 4 h (*n* = 6 cultures per group). (**E**) mRNA expression of *Mrc1* and *Igf2r* in the same cell types (*n* = 6 cultures per group). (**F**) Mrc1 detection by western blot of the same cell types. Canx western blot was performed as loading control. (**G**) ARSB activity in cell lysates from the same cell types after incubation with ARSB supplemented with (+) or without (−) M6P and mannose (Man) for 4 h (*n* = 6 cultures per group). Data are expressed as mean ± SD.

To address the question, if an intrinsic property of chondrocytes would hamper efficient rhARSB uptake, we isolated primary chondrocytes from wild-type and *Arsb^m/m^* mice and studied them *ex vivo*. Upon differentiation of the cells for 10 days in the absence or presence of rhARSB, followed by staining with alcian blue, we observed less chondrogenic differentiation in *Arsb^m/m^* cultures (Fig. 5A). By immunostaining for chondroitin 6-sulfate and the lysosomal marker protein Lamp1, we observed that the abundance of the latter was remarkably increased in *Arsb^m/m^* cultures. Moreover, this increase of lysosomal structures was less pronounced when the cells were cultured in the presence of rhARSB (Fig. 5B). Remarkably however, we did not detect specific staining with an ARSB-directed antibody in these cultures, which led us to compare rhARSB uptake in different cell types by western blot analysis after 4 h of rhARSB administration.

Consistent with our previous findings, rhARSB was efficiently endocytosed and delivered to lysosomes in *Arsb^m/m^* osteoclasts (17), since the proteolytically processed lysosomal form of the recombinant protein was detected in cell lysates (Fig. 5C). Efficient uptake and lysosomal delivery were also observed in primary osteoblasts, whereas the signal intensity in cell lysates of wild-type and *Arsb^m/m^* chondrocytes was remarkably lower when related to the signal intensity of the input protein. We therefore chose to determine intracellular ARSB activities in different cell types after 4 h of rhARSB administration. Here, we additionally included macrophages, fibroblasts and renal podocytes, the latter known to have decreased capacity to endocytose lysosomal enzymes (21). We found that ARSB activities in chondrocytes were as low as in podocytes and only 10% of the values determined in osteoclasts or osteoblasts (Fig. 5D). Since our previous analyses revealed that in osteoclasts, the uptake of M6P-containing rhARSB is dependent on mannose rather than that of M6P (17), we additionally screened the different cell types for the expression of two receptors facilitating endocytosis of lysosomal enzymes i.e. *Mrc1* (encoding the macrophage mannose receptor 1) and *Igf2r* (encoding the cation-independent M6P receptor) (22,23). Compared to osteoclasts, podocytes and chondrocytes displayed remarkably lower expression of *Mrc1*, but not of *Igf2r* (Fig. 5E). Remarkably, there was a clear correlation between rhARSB uptake efficacy and the expression level of *Mrc1* in all cell types analyzed, which was not observed for *Igf2r*.

After confirming the low expression of Mrc1 in podocytes and chondrocytes at the protein level (Fig. 5F), we addressed the question, if rhARSB endocytosis into different cell types would be blocked by addition of either mannose and/or M6P. We observed that rhARSB uptake into osteoclasts, osteoblasts, macrophages and fibroblasts was more efficiently blocked by mannose than by M6P (Fig. 5G). In contrast, the low uptake into podocytes and chondrocytes was only reduced by M6P. Taken together, these data reveal that Arsb is primarily endocytosed in a mannose-dependent manner. Moreover, the remarkable difference between bone-remodeling cell types and chondrocytes, in terms of rhARSB uptake efficiency and blockade by mannose/M6P, strongly suggests that reduced expression of *Mrc1* in chondrocytes underlies their decreased capacity to endocytose rhARSB and thus restricts efficacy of ERT in cartilage.

**Figure 6:**
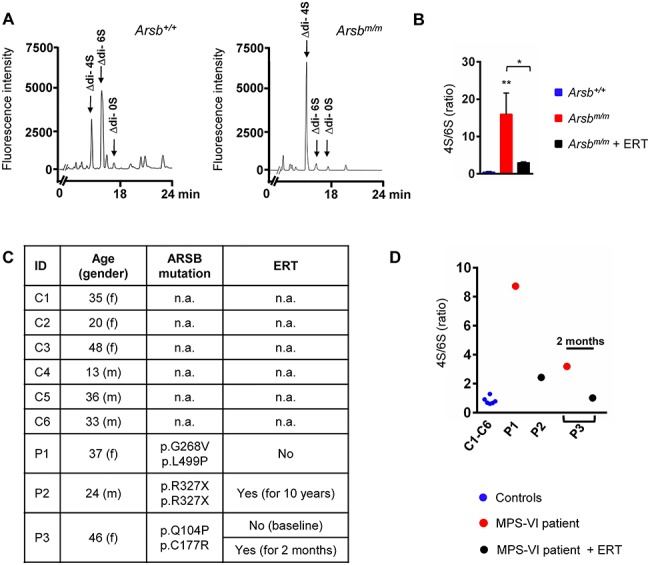
ERT affects urine concentrations of 4-sulfated chondroitin and dermatan sulfates. (**A**) Representative HPLC elution profiles of disaccharides after chondroitinase digestion of glycosaminoglycans from urine of 12-week-old wild-type (left) and *Arsb^m/m^* (right) mice. (**B**) Quantification of the ratio of 4-sulfated (4S) and 6-sulfated (6S) disaccharides in urine from wild-type, untreated and ERT-treated *Arsb^m/m^* mice at 12 weeks of age (*n* = 3 mice per group). (**C**) Information about control individuals (C) and MPS-VI patients (P), from which urine was analyzed for sulfated disaccharides. (**D**) Quantification of the ratio of 4-sulfated (4S) and 6-sulfated (6S) disaccharides in urine from the different individuals, as indicated. Data are expressed as mean ± SD. ^*^P < 0.05, ^**^P < 0.005 (one-way ANOVA).

### ERT reduces the concentration of 4-sulfated chondroitin and dermatan sulfates in urine

Having established the *Arsb^m/m^* mice as an excellent model of MPS-VI to study the efficacy of ERT, we finally addressed the question, if we could set up a valid biomarker approach to monitor patients receiving ERT. In fact, the majority of individuals with MPS-VI are currently screened by rather non-specific measurements of total urinary glycosaminoglycan levels (24–26), although the lack of Arsb, i.e. N-acetylgalactosamine-4-sulfatase, should cause a specific enrichment of 4-sulfated chondroitin and dermatan sulfates. We, therefore, performed chondroitinase digestion of urinary glycosaminoglycans from 12-week-old wild-type and *Arsb^m/m^* mice, and the resulting unsaturated ∆0S, ∆4S and ∆6S disaccharides were separated and quantified by HPLC (Fig. 6A). Here, we observed that the 4S/6S-ratio was more than 30-fold higher in untreated *Arsb^m/m^* mice than in wild-type littermates (Fig. 6B), demonstrating the accumulation of 4-sulfated chondroitin and dermatan sulfates in the urine of *Arsb^m/m^* mice. Most importantly, the 4S/6S-ratio was significantly reduced by ERT, which led us to perform a proof-of-principle analysis with human urine samples.

More specifically, we quantified the urinary 4S/6S-ratio in six control individuals and three patients with MPS-VI (Fig. 6C). Patient P1 was not treated by ERT and displayed a 13-fold increased 4S/6S-ratio compared to controls (Fig. 6D). In contrast, patient P2 had received weekly rhARSB infusion for the last 10 years and showed only a 3-fold increase of the 4S/6S-ratio. Given the high level of clinical heterogeneity between patients, the most important finding was related to patient P3. Here, the comparison of urine taken at baseline and 2 months after treatment initiation revealed a 3-fold reduction of the 4S/6S-ratio. These data essentially confirmed our expectation and imply that the quantification of 4-sulfated and 6-sulfated disaccharides in urine samples is a feasible and promising method to monitor disease progression and therapeutic effects in MPS-VI.

## Discussion

Lysosomal degradation of macromolecules is mediated by various enzymes, whose mutational inactivation typically results in accumulation of non-degraded material, which impairs the function of specific cell types (27). One relevant subgroup of lysosomal storage disorders are the mucopolysaccharidoses, which are caused by mutations in lysosomal enzymes required for the stepwise degradation of glycosaminoglycans (28). Based on the mutated enzyme, there are 11 different MPS disorders, some of them with a remarkable negative impact on the skeleton, i.e. dysostosis multiplex (29). Whereas patients with MPS-I, MPS-II or MPS-III are affected by abnormalities of the central nervous system, skeletal defects are predominant in MPS-VI. This difference is attributed to the fact that Arsb, the mutated enzyme in MPS-VI, is specifically involved in the degradation of chondroitin and dermatan sulfate, but not heparan sulfates, which accumulate in the brain and cause severe intellectual disability in other MPS disorders (9). Remarkably, however, although MPS-VI can be regarded as a skeletal disorder (30), the knowledge about the role of ARSB in specific skeletal cell types was very limited, as a corresponding deficient mouse model had not been analyzed on the basis of undecalcified histology and bone-specific histomorphometry (31,32).

Since two lysosomal enzymes, i.e. tartrate-resistant acid phosphatase (TRAP, Acp5) and cathepsin K (Ctsk), are commonly regarded as molecular markers of bone-resorbing osteoclasts (33,34), we have previously applied genome-wide expression analysis to identify additional lysosomal enzymes with a potential role in bone resorption. By utilizing this unbiased screening approach, we identified *Arsb* as a gene differentially expressed during osteoclastogenesis, which led us to perform in-depth skeletal phenotyping of a mouse model carrying an *Arsb* mutation (17). We observed that *Arsb^m/m^* mice display various aspects of dysostosis multiplex, i.e. impaired skeletal growth, craniofacial bone overgrowth or cervical cord compression, but also high bone mass due to impaired osteoclast activity. Most importantly, we observed strong lysosomal storage in all relevant skeletal cell types, demonstrating that Arsb is physiologically required for glycosaminoglycan degradation mediated not only by osteoclasts, but also by chondrocytes and osteoblast lineage cells. In a proof-of-principle experiment, we additionally found that the high trabecular bone mass phenotype of *Arsb^m/m^* mice was fully normalized by weekly injection of rhARSB given from the age of 12 weeks until the age of 24 weeks. However, this late-onset treatment strategy did not address the key question, if early initiation of ERT can prevent (not correct) the development of dysostosis multiplex, since this requires treatment initiation in growing mice.

Therefore, it was important to start the weekly rhARSB injection as early as 4 weeks of age and to study to what extent such delivery would prevent pathologies in different skeletal compartments and cell types of *Arsb^m/m^* mice. Our findings provided clear evidence that there was a near to complete prevention of bone-remodeling abnormalities, but essentially no influence on chondrocyte pathologies. In our opinion, these results are of high importance with respect to patient treatment. In fact, it is generally believed that the lack of a strong ERT effect on skeletal abnormalities of patients with MPS-VI and other MPS disorders is explained by inability of a systemically delivered enzyme to reach the respective cell types (15). Importantly, however, this does not apply to osteoclast and osteoblast lineage cells, since all of them are severely affected in *Arsb^m/m^* mice, but not in *Arsb^m/m^* mice receiving ERT. On the contrary, there was no therapeutic effect on chondrocytes, which are the primary cell types required for skeletal growth and joint integrity. Since the skeletal status of patients with MPS is typically documented by radiographic analysis, not by quantitative analysis of trabecular and cortical bone architecture, it is somehow expected that a major ERT influence is not easily identifiable, as the most evident aspects of dysostosis multiplex are chondrocyte-driven. In this regard, it is also important to state that our analysis of a bone biopsy from an ERT-treated patient, where we observed a specific enlargement of the chondrocyte population, fully confirmed our findings from *Arsb^m/m^* mice.

One key question that should be addressed in future studies on MPS-VI and other lysosomal storage disorders is how the chondrocyte delivery of recombinant lysosomal enzymes can be improved. Here, it is important to consider that cartilage, especially in the growth plate, is poorly vascularized, in contrast to the bone microenvironment (35). On the other hand, supported by proteomic analysis of porcine samples, the synovial fluid contains a large proportion (approximately 30%) of serum proteins, which are not produced locally (36). This raises the question, if the failure of ERT to correct chondrocyte defects in MPS-VI is fully explained by lack of vascularization. To address this question, we determined endocytosis and intracellular activities of rhARSB in different cell types after 4 h administration. We thereby observed that the efficacy of rhARSB uptake in cultured chondrocytes was the lowest among all analyzed cell types, suggesting that chondrocytes have an intrinsically low rhARSB uptake capacity. Notably, the expression of the M6P receptor (*Igf2r*) was comparable between the different cell types, whereas chondrocytes expressed the mannose receptor (*Mrc1*) at low level. Moreover, endocytosis of rhARSB into osteoclasts, osteoblasts and macrophages was strongly reduced in the presence of mannose, whereas the low rhARSB uptake into chondrocytes was only significantly reduced in the presence of M6P. On the other hand, as assessed by Lamp1 immunostaining, we observed that the lysosomal accumulation defects in cultured *Arsb^m/m^* chondrocytes was reduced despite the low rhARSB uptake efficacy. Although this suggests that even low levels of endocytosed rhARSB can correct the respective cellular deficits *in vitro*, our histologic analyses of ERT-treated *Arsb^m/m^* mice clearly show that such a correction does not take place *in vivo*. We therefore hypothesize that the lack of cartilage phenotype correction is explained by a combination of both, decreased vascularization as well as reduced mannose-dependent enzyme uptake into chondrocytes.

Importantly, most lysosomal enzymes used for ERT of lysosomal storage disorders, including rhARSB, are modified by M6P residues to initiate M6P receptor-dependent endocytosis and lysosomal delivery. On the other hand, the most common lysosomal storage disorder, i.e. Gaucher disease, can be treated with glucocerebrosidase that lacks M6P residues (37). In fact, the finding that a mannose receptor mediates the glucocerebrosidase uptake by macrophages, the mostly affected cell type in Gaucher disease, allowed to greatly improve the efficacy of ERT. Based on our data, we suggest that a failure of ERT to correct chondrocyte defects can be attributed, at least in part, to a low expression of *Mrc1* in chondrocytes, which cannot be compensated by M6P receptor-mediated endocytosis. We believe that these findings provide the basis for future studies to improve ERT in patients with MPS-VI. In this regard it is also important to state that mTORC1 hyperactivation has been shown to contribute to the growth defects of mice lacking ß-glucuronidase (a model for MPS-VII) or Arsb, thereby providing an alternative therapeutic approach to specifically correct chondrocyte defects in MPS disorders (38).

Besides studying the impact of ERT on skeletal development, growth and remodeling, we utilized the *Arsb^m/m^* mice in order to establish an improved biomarker readout to monitor treatment efficacy in MPS-VI. The majority of individuals with MPS-VI are currently screened by determination of urinary glycosaminoglycan levels. However, since infants naturally have high urinary glycosaminoglycan levels, this rather non-specific readout sometimes provides unclear results (24–26). Since the molecular function of Arsb is to hydrolyze one specific sulfate group in the stepwise degradation of chondroitin and dermatan sulfate, we took advantage of a previously established method, which enabled to detect chondroitin undersulfation and to monitor treatment in a mouse model of diastrophic dysplasia (39,40). More specifically, since about 90% of urinary glycosaminoglycans are chondroitin 4-sulfate and chondroitin 6-sulfate, we determined the sulfation pattern of disaccharides after chondroitinase digestion of urinary glycosaminoglycans. We observed an increase of the 4S/6S ratio in *Arsb^m/m^* mice, together with a clear reduction of this ratio in ERT-treated animals. Most importantly, we were able to demonstrate that this assay is not only valuable to diagnose human MPS-VI, but also to monitor treatment effects. Whether it is useful to apply this method for routine patient monitoring needs further investigation. However, with respect to clinical studies, it might be preferable to have a specific readout to control the functionality of the injected recombinant enzyme.

Taken together, our data obtained in a valid mouse model for MPS-VI, which were principally confirmed in a respective patient biopsy, allow us to conclude that defects in bone-remodeling cell types are prevented and corrected by systemic delivery of rhARSB, whereas chondrocyte defects are not. This knowledge should provide the basis to specifically improve chondrocyte delivery of the recombinant enzyme as well as patient monitoring.

## Materials and Methods

### Mice and treatment

*Arsb^+/m^* mice, carrying a mutation (c.379G > T) in exon 2 of the *Arsb* gene, were initially obtained from the Jackson Laboratory (#005598). Genotyping was performed by sequencing of a PCR amplicon (generated with the primers 5´-GCT ATA TCA CGG GCA CTA ATC C-3′ and 5′-TAT CGA ATC CTC GGC GTG T-3′). We only used littermate mice from heterozygous matings for comparative analyses and focused on female mice, since we have previously defined that there is no gender-related difference in phenotype (17). Generation of *Arsb^m/m^* mice and organ preparation were approved by the animal facility of the University Medical Center Hamburg-Eppendorf and by the ‘Behörde für Gesundheit und Verbraucherschutz’ (G14/068 and Org529). For ERT, *Arsb^m/m^* mice received weekly intravenous injection of rhARSB (Naglazyme®, BioMarin) at a dose of 1 mg/kg and in a volume of 150 μl. The treatment was started at 4 weeks of age and continued until the ages of 12 or 24 weeks, respectively. These experiments were approved by the animal facility of the University Medical Center Hamburg-Eppendorf and by the ‘Behörde für Gesundheit und Verbraucherschutz’ (G17/063).

### Skeletal analysis

Dissected skeletons were fixed in 3.7% PBS-buffered formaldehyde for 18 h, before they were stored in 80% ethanol. After initial assessment by contact X-ray, the lumbar vertebral bodies L1 to L4 and one tibia were dehydrated in ascending alcohol concentrations and then embedded in methylmetacrylate as described previously (41). Sections of 4 μm thickness were cut in the sagittal plane and stained by toluidine blue or von Kossa/van Gieson staining procedures. Histomorphometry was performed according to the ASBMR guidelines using the OsteoMeasure system (Osteometrics Inc.) (42). For μCT scanning we used the left femur and the skull that were scanned using a μCT 40 desktop cone-beam microCT (Scanco Medical) with a voxel size of 10 μm. Reconstructed slices were analyzed using the Scanco MicroCT software suite. Ultrastructural analysis by electron microscopy was performed according to standard protocols (17).

### Patient samples

The femoral head of a 30-year-old female patient with MPS-VI was removed during total hip arthroplasty, which was performed due to a combination of osteoarthritis and osteonecrosis secondary to hip dysplasia. The removed femoral head was fixed in 3.7% PBS-buffered formaldehyde for 18 h and then transferred into 80% ethanol for undecalcified histology. An age- and sex-matched control biopsy was obtained from a patient with osteoarthritis during total hip arthroplasty. Sections were stained by toluidine blue, and quantification of the lacunar area was performed using ImageJ. Urine was collected from three patients with MPS-VI and control individuals, as outlined in Fig. 6C.

### Cell culture

Primary chondrocytes were isolated by collagenase digestion from the rib cages of 10 days old mice and differentiated in the presence of 50 μg/ml ascorbic acid for 10 days. Alcian blue staining was performed according to standard protocols (43). Staining of lysosomes in fixed cultures was performed with a monoclonal anti-mouse Lamp1 antibody (clone 1D4B from Developmental Studies Hybridoma Bank, University of Iowa, 1:200) and a corresponding secondary antibody conjugated to Alexa Flour® 546. To detect chondroitin 6-sulfate a monoclonal antibody against chondroitin 6-sulfate ‘stubs’ (clone 3B3 from Amsbio, 1:20) was used following pre-digestion of chondroitin sulfate glycosaminoglycan chains with 35 mU chondroitinase ABC (Sigma-Aldrich) for 1 h at 37°C. Nuclei were stained with 4´,6-diamidino-2-phenylindole (DAPI). Primary osteoblasts were isolated by sequential collagenase digestion from the calvariae of 5 days old mice and differentiated in the presence of 50 μg/ml ascorbic acid and 10 mM ß-glycerophosphate for 10 days. Macrophages and osteoclasts were generated from bone marrow cells that were flushed out of the femora from 12-week-old mice. Cells were first cultured with 10 nM 1,25-dihydroxyvitamin-D3 (Sigma-Aldrich), and M-Csf (for macrophages) and M-Csf/Rankl (for osteoclasts) were added to a final concentration of 20 and 40 ng/ml, respectively starting at day 4. Functional assays were performed at day 7 of differentiation. The isolation of embryonic fibroblasts was performed as described elsewhere (44). Murine immortalized podocytes (a kind gift of Peter Mundel, Boston, MA, USA) were cultured in vented 25 mm^2^ tissue culture flasks for sensitive, adherent cells (Sarstedt) under permissive conditions: 32°C, 5% CO_2_, RPMI 1640 supplemented with 10% fetal calf serum, 15 mmol/l HEPES Buffer solution, 1 mmol/l sodium pyruvate, 10 U/ml IFN-gamma (PeproTech), 0.5 mg/ml G418 sulfate (Gibco). For differentiation, podocytes were cultured for 13 days under non-permissive conditions: 38°C, 5% CO_2_, RPMI 1640 supplemented with 10% fetal calf serum, 15 mmol/l HEPES Buffer solution, 1 mmol/l sodium pyruvate, 0.5 mg/ml G418 sulfate. Cell density was kept below 80–90% to allow process development.

### rhARSB uptake assays

To monitor cellular uptake of rhARSB, cultured cells were incubated for 4 h with serum-free Optimem™ medium containing Naglazyme® (10 μg/ml) in the presence or absence of 20 mM M6P and/or 80 mM mannose (both from Sigma-Aldrich). Cells were lyzed in 10 mM PBS (pH 7.4) containing 0.5% Triton X-100 and protease inhibitors for 30 min at 4°C. Aliquots of cell extracts (25 μg protein) or ARSB (50 ng) were separated by reducing SDS-PAGE and analyzed by western blotting with specific antibodies against human ARSB (R&D, #MAB4415, 1:500), mouse Mrc1 (R&D, #MAB2535, 1:500) or calnexin (Canx, Enzo Life Sciences, ADI-SPA-865, 1:1000), the latter serving as loading control. After incubation with secondary HRP-conjugated goat anti-rabbit IgGs, the immunoreactive bands were visualized by enhanced chemiluminescence. To measure ARSB activity, 5 mM 4-nitrocatechol-sulfate (Sigma-Aldrich) was used as a substrate in 10 mM Na-citrate (pH 5.5), 0.2% Triton X-100, 0.4% BSA and 10% NaCl. The incubation was stopped by addition of 0.4 M glycine/NaOH buffer (pH 10.4) after 17 h and the liberated 4-nitrocatechol was measured at 515 nm.

### Expression analysis

RNA was isolated using the RNeasyMini kit (Qiagen), and DNase digestion was performed according to manufacturer’s instructions. Concentration and quality of RNA were measured using a NanoDrop ND-1000 system (NanoDrop Technology). Expression analysis by qRT-PCR was performed using a StepOnePlus system and predesigned TaqMan gene expression assays (Applied Biosystems). *Gapdh* expression was used as an internal control. Relative quantification was performed according to the ΔΔC_T_ method, and results were expressed in the linear form using the formula 2^-ΔΔCT^.

### Glycosaminoglycan sulfation in urine

The success of ERT treatment was controlled by glycosaminoglycan sulfation analysis in urine as described previously (39,40). About 100 μl of urine was clarified by centrifugation and glycosaminoglycans were precipitated with 1/50 of 10% cetylpyridinium chloride overnight at 4°C. Samples were then centrifuged at 4°C and pellets were washed with 10% potassium acetate in 96% ethanol and with 96% ethanol, respectively. Pellets were digested at 37°C overnight with 30 mU of chondroitinase ABC (AMSBIO) and 30 mU of chondroitinase ACII (Sigma-Aldrich) in 0.1 M ammonium acetate buffer, pH 7.35. After lyophilization samples were derivatized with 2-aminoacridone (Life Technologies). HPLC chromatography was carried out with a C18 column (Prontosil 120–3-C18-ace-EPS 3.0, 4.6 × 200 mm, Bischoff) at room temperature and with a linear gradient of 0.1 M ammonium acetate, pH 8.0, (Buffer A) and 60:40 (v:v) acetonitrile with 0.1 M ammonium acetate, pH 8.0 (Buffer B). Eluates were monitored using a fluorescence detector (2475 Multi λ Fluorescence Detector, Waters) with excitation and emission wavelengths of 425 and 525 nm, respectively.

### Statistics

All data are presented as mean ± standard deviations. For the comparison of two groups statistical analysis was performed using unpaired, two-tailed Student’s t test (Microsoft Excel®). For the comparison of multiple groups we used ANOVA (Graph Pad Prism®). In both cases differences with p-values below 0.05 were considered statistically significant.

### Study approval

Generation and treatment of *Arsb^m/m^* mice and organ preparation were approved by the animal facility of the University Medical Center Hamburg-Eppendorf and by the ‘Behörde für Gesundheit und Verbraucherschutz’ (G14/068, G17/063and Org529).

Procedures carried out with human subjects were in compliance with the Helsinki Declaration. All human samples were taken with written informed consent of the patients.
